# Functional Characterization of the *Saccharomyces cerevisiae* Equilibrative Nucleoside Transporter 1 (ScENT1)

**DOI:** 10.3390/molecules23040732

**Published:** 2018-03-22

**Authors:** Rebba C. Boswell-Casteel, Jennifer M. Johnson, Franklin A. Hays

**Affiliations:** 1 Department of Biochemistry and Molecular Biology, University of Oklahoma Health Sciences Center, Oklahoma City, OK 73104, USA; rcasteel86@gmail.com (R.C.B.-C.); Jennifer-M-Johnson@ouhsc.edu (J.M.J.); 2 Stephenson Cancer Center, University of Oklahoma Health Sciences Center, Oklahoma City, OK 73104, USA; 3 Harold Hamm Diabetes Center, University of Oklahoma Health Sciences Center, Oklahoma City, OK 73104, USA

**Keywords:** nucleoside transport, transporter, nucleoside, nucleotide, membrane protein

## Abstract

Equilibrative nucleoside transporters (ENTs) are polytopic membrane transporters responsible for the translocation of nucleosides, nucleobases—to a lesser extent—and nucleoside analog therapeutics across cellular membranes. ENTs function in a diffusion controlled bidirectional manner and are thought to utilize an alternating access transport mechanism. However, a detailed understanding of ENT function at the molecular level has remained elusive. ScENT1 (formerly known as Function Unknown Now 26 or FUN26) is the only known ENT ortholog endogenously expressed in *S. cerevisiae*, and a proteoliposome assay system was used to study homogenously overexpressed and purified ScENT1 (wildtype relative to L390A and F249I mutants). L390 and F249 are highly conserved residues and were found to alter transporter function. L390A produced a reduction of mean transport activity while F249I increased mean substrate translocation relative to wildtype protein. However, both mutations resulted in transport of UTP—a novel gain of function for any ENT. These residues were then mapped onto an ab initio model of FUN26 which suggests they function in substrate translocation (L390) or cytoplasmic gating (F249). Furthermore, wildtype, L390A, and F249I were found to be sensitive to the presence of alcohols. Ethanol attenuated ScENT1-mediated transport of uridine by ~50%. These findings further demonstrate functional similarities between ScENT1 and human ENT isoforms and support identification of FUN26 as ScENT1, the first ENT isoform in *S. cerevisiae*.

## 1. Introduction

Nucleoside transporters are fundamental contributors to nucleoside physiology, pathophysiology, and the beneficial exploitation of nucleoside analog therapeutics. Nucleosides serve as metabolic precursors in de novo nucleic acid synthesis, metabolic precursors of energy metabolism (e.g., ATP and GTP), and as ligands for purinergic receptors (e.g., adenosine and inosine) [1,2]. Nucleoside and nucleobase analogs also represent important classes of antineoplastic agents and antiviral therapeutics [3]. Activity of many of these hydrophilic compounds relies upon their entry into intracellular metabolic pathways to exert their effectiveness. Thus, transport through cellular membranes is an essential component of therapeutic efficacy for nucleoside and nucleobase derived therapeutics. 

Equilibrative nucleoside transporters (ENTs) are polytopic integral membrane proteins (IMPs) that regulate the plasmalemmal flux of purine and pyrimidine nucleosides and nucleobases, but not nucleotides [3]. In addition to endogenous ligands, ENTs modulate efficacy for a variety of FDA/EMA approved therapeutics (e.g., anticancer, antiarrhythmia, antihypertensive, and antiviral medications), and ENTs are also known biomarkers for drug efficacy in the treatment of certain cancers [4,5]. ENTs have also been implicated in modulating seizure activity by regulating adenosine flux [6], and in seizures associated with alcohol withdrawal syndrome [7,8]. Functionally, ENTs are bidirectional, facilitative transporters that utilize concentration gradients of transportable substrates to regulate permeant influx and efflux across membrane bilayers. There are four human ENT isoforms (hENT1–4), each having 11 predicted transmembrane domains (TMDs) and large hydrophilic loops at either the N-terminus (hENT3–4) or between TMD5 and TMD7 (hENT1–4) [9]—a predicted topology that is similar for members of the Major Facilitator Superfamily (MFS) [10]. Currently, there are no molecular structures for any member of the ENT family and, until recently, ENTs were resistant to characterization in purified form. ENT7 from *Arabidopsis thaliana* [11] and Function Unknown Now 26 (FUN26) from *Saccharomyces cerevisiae* [12] (henceforth referred to as ScENT1) are the only ENT family members to undergo detergent extraction from the lipid bilayer and retain function in a purified state. 

ScENT1, the only identified hENT1–4 ortholog found in *Saccharomyces cerevisiae*, is predicted to have: (1) 11 TMDs; (2) an extramembrane loop connecting TMDs 6 and 7; and (3) a hydrophilic N-terminal domain [12,13]. Functional characterization in proteoliposomes demonstrated that ScENT1 is a broadly selective, high affinity, nucleoside and nucleobase transporter, with positional sensitivities to modifications at the C(2′)- and C(5′)-positions of the ribose ring [12]. Since ScENT1 is predominantly localized to yeast vacuoles [13,14], the observed broad substrate selectivity would suggest that ScENT1 is involved in salvaging nucleosides and nucleobases from inside vacuoles and recycling them to cytoplasmic pools. In these studies, ScENT1 demonstrated a unique substrate transport profile when compared to human ENTs. However, mutagenesis data revealed that non-synonymous single nucleotide polymorphisms (SNPs), found in the coding region of hENT3 (G463, ScENT1) [15], and a previously characterized mutation (G216, ScENT1) found in hENT1 [16] had similar functions when studied in ScENT1 [12]. While ScENT1 is functionally distinct from previously characterized ENTs, it retains overlapping structural features with hENT1 and hENT3—therefore, further characterization of ScENT1 in the purified state would advance the understanding of ENT molecular function and facilitate structure determination efforts for this difficult family of IMP transporters. 

In the present study, we utilized an in vitro proteoliposome (PL) transport assay system to functionally characterize ScENT1 point mutations using purified protein. We also utilized this system to assess what role alcohols may play in modulating ScENT1 transport activity. Previous data suggests that ENT proteins function as direct mediators of ethanol sensitivity [17,18,19]. Point mutations were selected based upon known hENT SNPs associated with disease (e.g., G216 and G463) or conserved sidechain characteristics (e.g., L390 and F249) relative to predicted structural features (e.g., transmembrane helices). The L390A and F249I mutations were selected as they reside in strictly conserved sites in the ENT family located in predicted TMDs (TMD6 for F249 and TMD8 for L390). Conserved aromatic residues located at the distal TMD ends can serve key functional or regulatory roles in integral membrane transporter function [20]. Point mutation selection was also guided by ab initio structural modeling of ScENT1 as reported below. In addition, functional studies using human lymphocytes [19], murine lymphoma cells [19], and hybrid neuroblastoma and glioma cells [21] suggest that hENT1 transport activity is modulated by ethanol. Given the functional overlap between ScENT1 and previously identified ENTs, we hypothesized that ethanol would modulate ScENT1-mediated substrate transport activity following reconstitution into PLs. An advantage of using PLs is the ability to control lipid bilayer composition and luminal buffer conditions. Data obtained in the present study demonstrates that (1) the L390A and F249I mutations are capable of modulating substrate influx and altering the overall substrate transport profile; (2) residues L390, and G463, appear to contribute to the substrate translocation pore while G216 contributes to structural stability of the protein and F249 may regulate cytoplasmic gating; (3) L390A and F249I ScENT1 mutants are capable of low levels of [^3^H]-UTP transport—the first demonstration of ENT-mediated nucleotide transport; (4) ScENT1-mediated uridine transport is attenuated in the presence of ethanol; and (5) the overall substrate transport profile of ScENT1 is altered by alcohols. Thus, this work advances the molecular understanding of ENTs and provides direct evidence for ethanol modulation of substrate transport using a purified ENT family member.

## 2. Results

### 2.1. Nucleoside, Nucleobase and Nucleotide Uptake by the L390A and F249I Mutant Proteoliposomes

**Figure 1:**
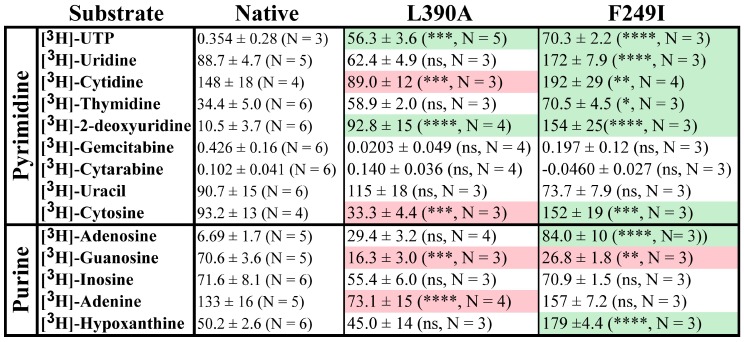
Comparison of the uptake of radiolabeled substrate by Native ScENT1 and L390A and F249I mutants. Substrates have been classified based on containing a pyrimidine or purine nucleobase and data represents the mean substrate uptake (pmol substrate/mg of ScENT1) for each substrate tested at 100 nM final concentration in the assay sample mixture, *N*-values are indicated next to each substrate in the figure. PLs were incubated with radiolabeled substrate for 3.5 h followed by vacuum filtration onto membranes. Error bars represent the S.E.M. Statistical significance of the L390A and F249I PLs was determined by comparing the mean substrate uptake for each individual substrate to the mean substrate uptake of the respected Native PLs using two-way analysis of variance and Dunnett’s multiple comparison test [^3^H]-cytarabine uptake for the Native PLs was used as a reference. Statistically significant changes are denoted in red (decrease) or green (increase) relative to Native ScENT1. Negative control PLs and substrate specific activity are included in the pmol substrate/mg ScENT1 calculation for each sample. ns, not significant; * *p* < 0.05; ** *p* < 0.01; *** *p* < 0.001; **** *p* < 0.0001.

In the present studies, substrate uptake by PLs is determined post vacuum filtration, following a 3.5 h incubation with 100 nM tritiated substrate, by quantifying the radioactive signal on borosilicate filters using liquid scintillation counting. In an effort to preserve hydrophobic side chain characteristics, while probing functional alterations, a conservative isoleucine mutation was made at F249, while L390 was mutated to alanine. It has been repeatedly demonstrated that ENTs are capable of distinguishing between nucleosides and nucleotides, as no ENT ortholog has demonstrated transport of substrates containing phosphorylation on the C(5′) position of the ribose sugar [22,23]. However, when the L390A and F249I mutations were examined, statistically significant [^3^H]-UTP transport was observed (Figure 1) for both. Further examination of the mean transport profiles for both mutations revealed that the F249I mutant generally increased transport, while the L390A mutation resulted in a reduction of mean transport. Specifically, F249I increased transport for [^3^H]-uridine, [^3^H]-cytidine, [^3^H]-thymidine, [^3^H]-deoxyuridine, [^3^H]-cytosine, [^3^H]-adenosine, and [^3^H]-hypoxanthine (fold increase ranged from 1.3 to 15), while L390A decreased transport for [^3^H]-cytidine, [^3^H]-cytosine, [^3^H]-guanosine, and [^3^H]-adenine (fold decrease ranged from 1.7 to 8.8, Figure 1). Both mutations resulted in strong increases in [^3^H]-deoxyuridine (15X, F249I; 8.8X, L390A) transport and decreases in [^3^H]-guanosine transport (2.6X, F249I; 4.3X, L390A, Figure 1). Additionally, F249I had a greater effect on pyrimidine substrates relative to purine substrates (Figure 1). 

**Figure 2:**
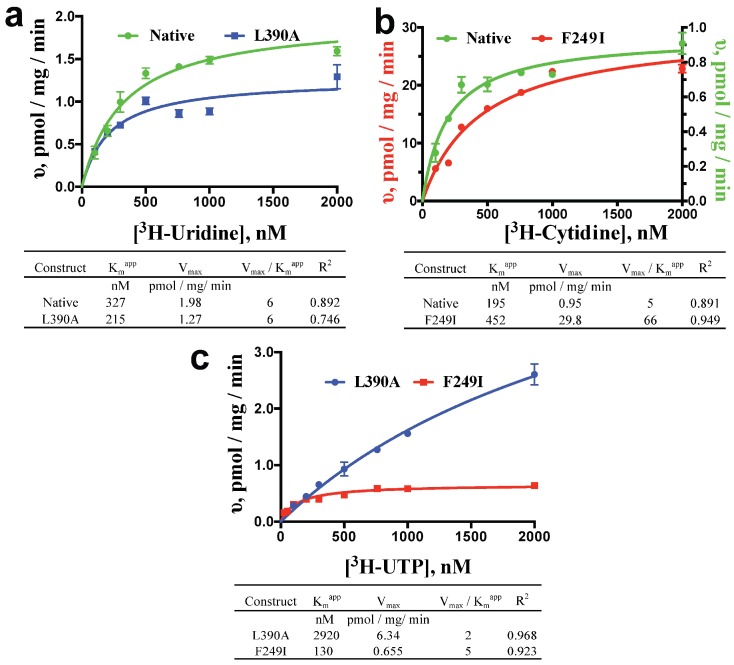
Kinetics of [^3^H]-uridine (**a**), [^3^H]-cytidine (**b**), and [^3^H]-UTP (**c**) by Native and Mutant ScENT1-containing PLs. Substrate influx was measured at 10, 20, 50, and 60 min using substrate concentrations ranging from 26 nM to 2000 nM for most substrates tested. The slope of the linear time course for each substrate concentration was then fit directly using nonlinear regression to determine initial uptake rates for each substrate concentration. These values, in pmol/mg/min, were then plotted relative to substrate concentration and fitted directly using nonlinear regression and a simple steady-state Michaelis-Menton model. Resultant values for K_m_^app^, V_max_, transport efficiency (V_max_/K_m_^app^), and *R*^2^ are listed in the tables below each graph. N = 3 for all L390A (blue) and F249I (red) trials. Native (green) trails are N = 2 for cytidine, and N = 4 for uridine.

Apparent K_m_ (K_m_^app^) and V_max_ values were determined by measuring substrate influx at various time points and substrate concentrations. Initial uptake rates were linear across 60 min for each substrate concentration tested (Figure S1), and saturable at higher concentrations (Figure 2). The observed linearity across 60 min may reflect a low copy number of active transporter per PL vesicle, though quantitative ScENT1 insertion was observed up to 400 μg which corresponds to ~3% of the total lipid bilayer volume. ScENT1 is presumed to interact with a single permeant molecule during each transport cycle. Therefore, kinetic data was assessed using a simple steady-state Michaelis-Menten model (Figure 2). Kinetic analysis revealed that L390A only slightly decreased the K_m_^app^ (higher affinity) and decreased the V_max_ (slower transport) for [^3^H]-uridine, resulting in no change in transport efficiency when compared to native ScENT1 (Figure 2a), and F249I moderately increased the K_m_^app^ (lower affinity) and drastically increased the V_max_ (faster transport) for [^3^H]-cytidine, yielding an increase in transport efficiency when compared to native ScENT1 (Figure 2b). The observed kinetics for the L390A and F249I mutations correlated to the observed changes in the mean substrate uptake for [^3^H]-uridine and [^3^H]-cytidine. When the kinetics of [^3^H]-UTP transport were examined, L390A exhibited a 2920 nM K_m_^app^, a V_max_ of 6.34 pmol/mg/min, and yielded an overall transport efficiency of 2, and F249I resulted in a K_m_^app^ of 130 nM, a V_max_ of 0.665 pmol/mg/min, which resulted in an overall transport efficiency of 5 (Figure 2c). Indeed, the F249I mutant had a higher mean substrate uptake when compared to the L390A mutant (Figure 1). It should be noted, to more accurately determine L390A [^3^H]-UTP transport activity (V_max_ and K_m_^app^) would require testing higher [^3^H]-UTP concentrations to obtain saturable conditions. The current experiments were limited by specific activity and concentration of the [^3^H]-UTP stock solution. As such, the stated V_max_ and K_m_^app^ have been extrapolated from the observed transport data (Figure 2c). 

### 2.2. Ab Initio Modeling of FUN26

**Figure 3:**
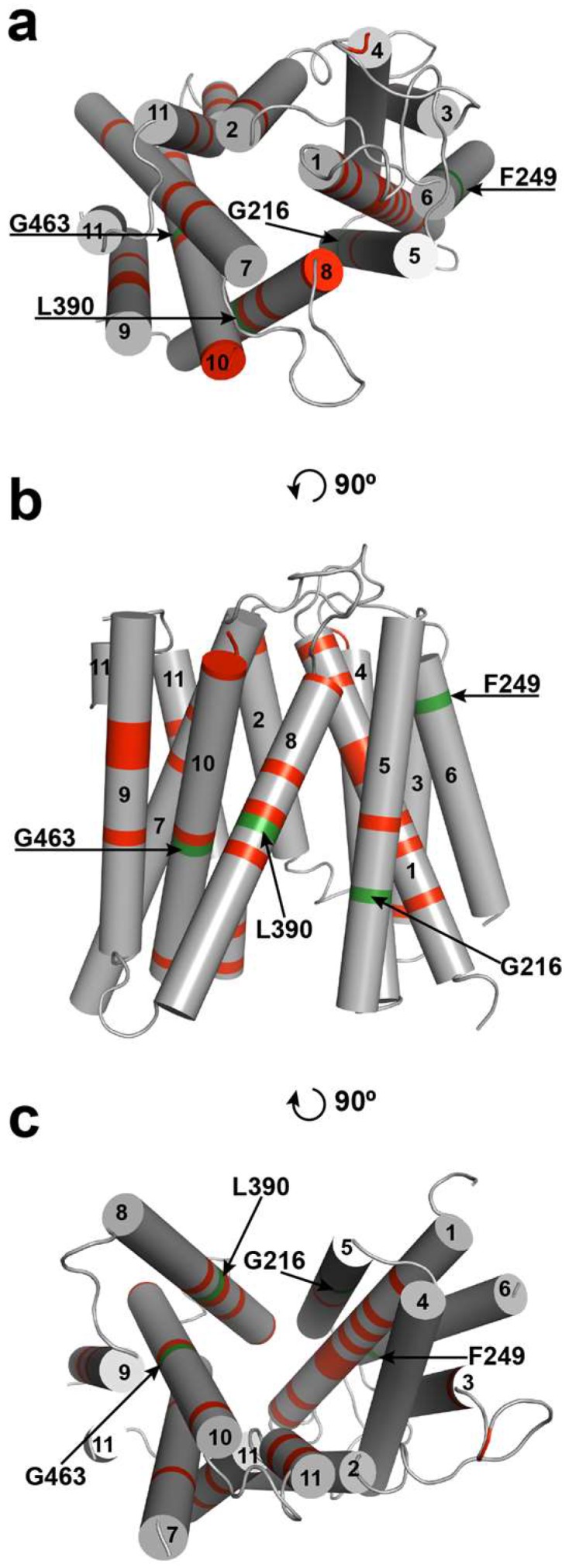
Inward-open model of ScENT1 structure. Strictly conserved residues are denoted in red (~16% of total sequence). Residues functionally characterized using ScENT1 PLs are denoted in green and highlighted by arrows. Helix numbering is shown as 1–11 beginning with the N-terminus, and helix 11 is shown as discontinuous. (**a**) top-down view of structure (cytoplasmic side), (**b**) side view—lipid bilayer not depicted, and (**c**) bottom-up view of structure (luminal side). Panel **a** and panel **c** are simple rotations of 90 degrees relative to **b**.

Currently, no ENT molecular structures have been determined using crystallographic, cryo-electron microscopy, or NMR methodologies. Since structural commonality has been noted between other MFS transporters, namely *E. coli* lactose permease (LacY) [24] and glycerol 3-phosphate transporter (GlpT) [25], a putative ab initio tertiary structural model of ScENT1 was developed based on bacterial sugar transporters with known crystallographic structures (Figure 3). The ScENT1 model depicts 11 TMDs organized similarly to the recognized canonical MFS fold [26], with TMDs 1, 4, 7, and 10 positioned in the center of the transporter; TMDs 2, 11, 5, and 8 positioned surrounding central core helices; and TMDs 3, 6, and 9 located on the outside protein periphery. In our model, ScENT1 is predicted to adopt an inward-open conformation, and is consistent with prior crystallographic observations for other members of the MFS [10] in that the inward-open conformation is a minimal energy configuration and well represented amongst known MFS transporter structures available in the PDB. While the model predicted the 11 TMDs to approximate an arrangement similar to other MFS proteins, it was unable to model the large extramembrane domain located at the N-terminus between TMDs 6 and 7. These regions were shown as large unstructured regions and were subsequently removed from the model to preserve clarity as they have no significant sequence homology to known structures. 

Next, the L390A and F249I mutations were mapped onto the ab initio model of FUN26 in order to better interpret potential structural implications of functional results from the PL studies (Figure 3). F249 is predicted to reside at the distal end of TMD6, while L390 is located in the middle of TMD8. Previous studies have shown that aromatic residues within TMDs may contribute to gating [27,28] or substrate selectivity [29,30]. It has also been shown that residues positioned on TMDs outside of central core helices facing the transport pathway may participate in substrate binding [31]. In addition to the F249I and L390A mutations, G216A and G463A were also mapped onto the ab initio structure. G216A and G463A were previously identified as mutations that resulted in loss of [^3^H]-uridine transport (G463A) or expression (G216A) [12]. G216 is strictly conserved in all ENTs (Figure S2) and has been shown to be essential for ENT1-mediated transport [16]. It also aligns in the region of G184 in ENT1, which has been shown to be involved with targeting ENT1 to the plasma membrane [16]. Additionally, G463 is strictly conserved in all putative ENTs, and corresponds to a non-synonymous SNP found in ENT3 that is associated with H and PHID syndrome (human genetic disorders), and has been found to abrogate ENT3 mediated transport in a manner that is independent of protein abundance or cellular localization [15]. These residues map onto TMD5 (G216) and TMD10 (G463), respectfully. 

ENTs are promiscuous transporters that demonstrate broad selectivity for nucleosides, nucleobases, and various nucleoside or nucleobase analogs [3]. This insight has been gained through multiple functional studies using tissue culture, oocytes, crude membrane preparations, and proteoliposomes aimed at assessing ENT function. However, little focus has been centered on the substrates themselves. We recently reported that ScENT1 was sensitive to C(2′)-ribose modifications [12]. Therefore, we analyzed thymidine, deoxyuridine, cytidine, uridine, cytarabine, and gemcitabine using Gaussian 09 to determine if any underlying chemical attributes at the C(2′) position of the ribose ring were directly contributing to substrate specificity. Partial charge distributions (1 to −1) were similar for the two transportable substrates (uridine and cytidine) and cytarabine, a stereoisomer of cytidine (Table S1). Removal of the C(2′)-hydroxyl group produced a partial charge distribution at the C(2′)-ribose increasing negativity for deoxyuridine and thymidine when compared to the transportable substrates, but remained similar between the two C(2′)-deoxy compounds (Table S1). The most notable difference was caused by the electron withdrawing C(2′)-difluorine substitution on gemcitabine (Table S1) causing Mulliken charges to become increasingly positive (~0.80 vs. ~0.15). When the electrostatic surface potentials were examined, the stereoisomer cytarabine was observed to have an increased positive potential centered above the ribose ring (Figure S3) that was absent in transportable substrates and other substrates with C(2′)-ribose modifications (Figure S3). 

### 2.3. ScENT1-Mediated Transport is Attenuated by Alcohols

**Figure 4:**
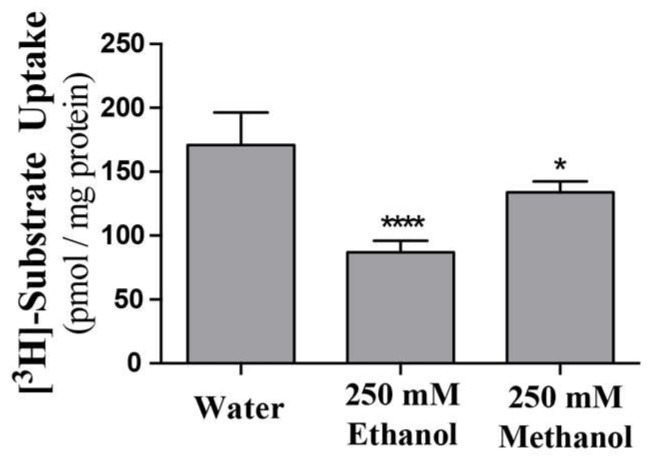
Alcohol attenuation of ScENT1-mediated uridine transport. Ethanol and methanol attenuation was determined by pre-incubating (–OH) PLs with 250 mM of the denoted alcohol or an equal volume of water for 15 min, then incubating with 100 mM of [^3^H]-uridine for an additional 3.5 h followed by vacuum filtration onto membranes. Error bars represent the S.E.M. of N = 3 independent experiments. Negative control PLs and substrate specific activity are included in the pmol substrate/mg ScENT1 calculation for each sample. Statistical significance was determined using a one-way analysis of variance and Dunnett’s multiple comparison tests (* *p* < 0.05; **** *p* < 0.0001).

**Figure 5:**
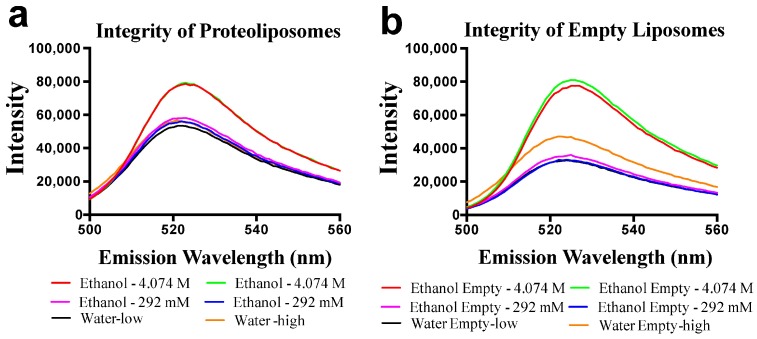
Membrane integrity is maintained in the presence of ethanol and methanol. PLs (**a**) and empty liposomes (**b**) were loaded with 50 mM CF prior to extrusion. Equal volumes of alcohol or water (control) were added to each sample of PLs (**a**) or empty liposomes (**b**). Each sample was then mixed, cuvettes capped off, and emission was monitored between 500 nm and 560 nm. Red, ethanol 4.074 M; magenta, ethanol 292 mM; green, ethanol 4.074 M; blue, ethanol 292 mM; black, water volume corrected to match 292 mM ethanol volumes; yellow, water volume corrected to match 4.074 M ethanol volumes.

Previous data suggests, or infers, that ENT’s may be directly inhibited by ethanol or other alcohols [17,18,19]._ENREF_29 Using purified ScENT1 reconstituted into PLs, we observed attenuated ScENT1-mediated transport of [^3^H]-uridine (100 mM, Figure 4) in the presence of ethanol (250 mM) and, to a lesser extent, methanol (250 mM). This is the first demonstration of direct alcohol-mediated transport modulation using a purified ENT. This was confirmed after verifying that the observed decrease in [^3^H]-uridine transport was not due to disrupting the artificial membrane environment of the PLs or empty liposomes containing 50 mM CF in the luminal volume (Figure 5 and Figure S4). In fact, ethanol did not have a substantial impact on PL integrity until it surpassed ethanol concentrations greater than 1.1 M and the integrity of empty liposomes was intact until ethanol surpassed 400 nM (Figure S4). A more comprehensive study is required to determine what affect, if any, lower ethanol concentrations have on ScENT1 and hENT transport activity.

**Figure 6:**
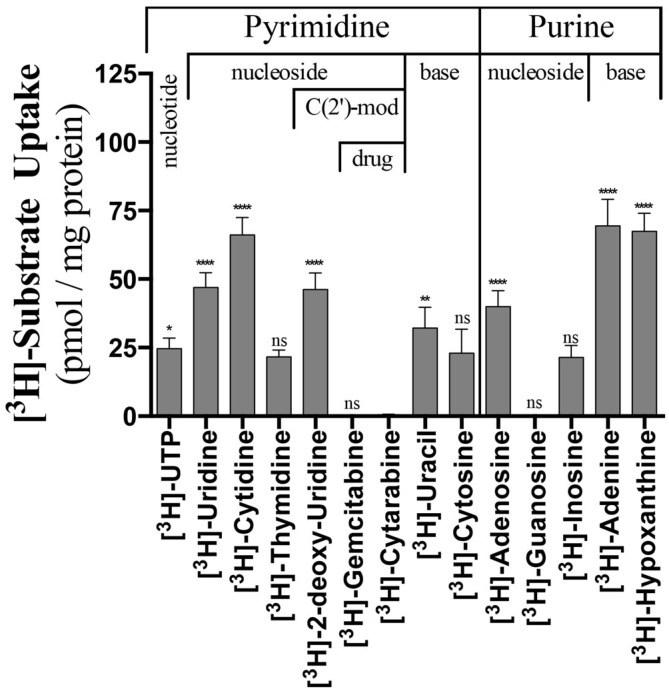
Uptake of radiolabeled substrates into ScENT1 (–OH) PLs. All substrates tested in this study were tested in PLs prepared in the absence of residual alcohols and are divided into pyrimidine and purine classifications further separated into nucleotide, nucleoside, and nucleobase categories with C(2′)-modification noted. Data represents the mean uptake from N = 9 (Native) and N = 3 (L390A and F249I) independent observations for each substrate tested (100 nM final concentrations), and *N*-values are presented on the graph. PLs were incubated with substrate for 3.5 h prior to being vacuumed filtered onto membranes. Error bars represent the S.E.M. Statistical significance was determined by comparing the mean substrate uptake (pmol substrate/mg ScENT1) for substrates relative to [^3^H]-cytarabine uptake by PLs using ordinary one-way analysis of variance and Dunnett’s multiple comparison test. Negative control PLs and substrate specific activity are included in the pmol substrate/mg ScENT1 calculation for each sample (see Methods). ns, not significant; * *p* < 0.05; ** *p* < 0.01; **** *p* < 0.0001.

Considering this new observation that alcohols modulate ScENT1 uridine transport activity, we sought to remove all reagents containing primary or secondary alcohols in light that they may alter substrate specificity and transport. Our standard approach for protein and PL preparations utilize protease inhibitors suspended in 2-propanol, the reducing agent β-mercaptoethanol, and glycerol for protein stability. We developed protein production and PL preparation protocols that removed all sources of residual alcohols. Complete removal of residual alcohols (2-propanol and β-mercaptoethanol) from the purification scheme did not alter protein stability or yield. However, glycerol was required during the initial purification stages (pre-SEC) to maintain protein stability [32]. We then retested the alcohol free (–OH) ScENT1 preparations (wildtype and mutant) to look for alterations in substrate transport properties (Figure 6). Interestingly, ScENT1 (–OH) is gained [^3^H]-UTP, [^3^H]-deoxyuridine, and [^3^H]-adenosine transport activity—substrates not transported in the presence of residual alcohols. A loss of significant transport is observed for [^3^H]-thymidine, [^3^H]-cytosine, and [^3^H]-guanosine (Figure 6). The transport profile obtained in the absence of residual alcohols was altered when compared to ScENT1 expressed in oocytes [13], but ScENT1 (–OH) is in agreement that it now transports [^3^H]-adenosine—a substrate known to be transported by all ENTs [3]. Surprisingly, we see that ScENT1 (–OH) is capable of transporting [^3^H]-UTP. ENT-mediated transport of substrate containing a C(5′)-ribose triphosphate modification is a novel observation [3]. When we compared ScENT1 (+OH) to ScENT1 (–OH) (Figure S5a) the +OH PLs have a statistically significant increase in mean substrate uptake for most substrates tested. The increase in mean substrate transport for (+OH) preparations also held true for the F249I mutants, while the L390A mutant transport profile was less varied (Figure S5b,c). 

Next, the L390A (–OH) and F249I (–OH) mutants were compared to native ScENT1 (–OH) containing PLs. F249I (–OH) still results in a general increase in mean substrate uptake, but L390A (–OH) now gives a more mixed profile (Figure S6). F249I (–OH) also shows a strong preference for increasing transportability of purine substrates tested in this study (Figure S6). In contrast, L390A (–OH) preferentially increases the uptake of pyrimidine nucleosides (Figure S6).

## 3. Discussion

ENT proteins modulate transmembrane flux for a broad range of nucleoside and nucleobase-derived small molecules, including human therapeutics, yet the underlying molecular mechanism and structure are unknown. This gap in understanding is derived from difficulties associated with overexpressing, and purifying, functionally active full-length ENT proteins [3,33,34]. Only recently has it been reported that ENTs are capable of being solubilized by detergents and functionally analyzed in purified form using proteoliposomes [12] (*S. cerevisiae* FUN26) or by microscale thermophoresis [11] (*A. thaliana* ENT7). The present study utilizes purified wildtype, and mutant, ScENT1 protein reconstituted into defined liposome systems to further assess how this family functions at the molecular level. Three conserved residues (L390, F249 and G216) and a known human SNP (G463) were selected based upon sequence alignments (Figure S2) and computational modeling (Figure 3) [27]. 

L390 is a highly conserved hydrophobic residue in TMD8 and positioned in the middle of an NXXD(X,L)XGR motif that is strictly conserved in hENT1-3, bovine ENT1-3, rat ENT1-3, mouse ENT1-3 and LdNT1.1 (Figure S2). The observation that L390A was able to alter the substrate profile and generally reduce mean substrate transport, combined with its positional location in the ab initio model, suggests that it is directly involved with substrate translocation through the protein interior. While TMD8 of MFS transporters is positioned outside of the core helices and is generally thought to mediate an interface between N-terminal and C-terminal domains [10], sidechains that project into the transport pathway can participate in substrate binding. F249 is a strictly conserved residue in TMD6. Substitution of the F249 aromatic sidechain with a hydrophobic isoleucine residue preserved transporter function, albeit with higher mean uptake and an altered transport profile. TMD6 has been previously implicated with substrate binding in Mhp1 [35], nucleobase transport activity in rat ENT2 [36], and part of a π interaction network [20] in sodium symporters. Given that aromatic residues distally placed on helices have been shown to be involved with transporter opening and closing, or “gating”, mechanisms [20,27,28], F249 of ScENT1 may contribute to cytoplasmic gating by stabilizing the cytoplasmic loop region [12]. Additionally, F249 may form stabilizing CH–π or cation–π interactions with the interhelical loop or with adjacent helices that aid in transitioning between conformations. Furthermore, a linker domain consisting of 30–100 amino acids links TMD6 to TMD7 and connects the N-terminal and C-terminal regions of ScENT1. Previous studies suggest this region forms an unstructured loop [26,27,28], though no structures have been determined. In the present study, this linker region and the large N-terminal domain were removed in ab initio models to provide clarity. Thus, F249 may also play a role in modulating TMD6 dynamics and overall ScENT1 conformational variability. 

Residue G216 is positioned outside of the core helices in TMD5 (Figure 3). This transmembrane helix has been associated with nucleobase transport in rat ENT2 [36], protein targeting to the plasma membrane [16], and transporter function [37]. We previously observed that the G216A mutation resulted in significant attenuation of ScENT1 expression [12]. This would suggest that the G216 position is essential for proper protein folding or membrane targeting. However, like L390, G216 maps onto the region suspected of interacting directly with substrate, and if the sidechain projects into the translocation pore it may be involved with substrate binding. Likewise, we propose that G463 is also involved in direct substrate binding interactions. G463A is a known human SNP found in hENT3 and associated with the autosomal recessive disorders H syndromes, pigmentary hypertrichosis and non-autoimmune insulin-dependent diabetes mellitus (PHID) syndrome, familial Rosai-Dorfman disease, and histiocytosis-lymphadenopathy plus syndrome [15]. These syndromes are associated with pancreatic exocrine insufficiency, stunted growth, pubertal delay, persistent inflammation, and cardiomyopathy. G463 is conserved in ScENT1 (Figure S2) and predicted to reside in TMD10 according to the ab initio model. TMD10 has been shown to be involved in substrate binding and release [10,31] and an intracellular gating mechanism in LdNT1.1 [28]. We previously reported that, like G463′s hENT3 counterpart, even a minor mutation to alanine results in the loss of uridine transport, while maintaining expression levels comparable with native ScENT1 [12]. These results, combined with the location of G463 on the ab initio model, would suggest that G463 is directly involved with the binding of substrate. However, any interpretation of mutagenesis data in the absence of an atomic structure must be done so with caution as point mutations may disrupt protein structure (either locally or distally). 

Using ScENT1 reconstituted PL assays [12], these data demonstrate that ScENT1-mediated transport of uridine is attenuated in the presence of ethanol (Figure 4). The reduction in mean transport does not result from decreased membrane integrity (Figure 5 and Figure S4). It is unknown if ethanol attenuation is the result of specific protein interactions with either the cytoplasmic or luminal face of ScENT1, the translocation pore itself, or changes in PL fluidity or lipid dynamics. Protein insertion into the artificial membrane environment is stochastic with ENT’s functioning as bidirectional transporters along a concentration gradient. 250 mM ethanol attenuates ScENT1-mediated uridine transport by approximately 50%, suggesting either facio-specific binding interactions or conformation-dependent binding. When residual primary and secondary alcohols were removed from protein purification steps, a shift in the substrate transport profile was observed allowing for passage of larger molecular volume substrates (e.g., UTP and adenosine as shown in Figure 6 and Figure S5a).

Given that wildtype ScENT1 has been observed to have high affinity and broad selectivity for substrates [12], it is expected to play a role in recycling nucleosides and nucleobases between the vacuolar lumen and cytosol. Furthermore, the ability of ethanol [250 mM or 1.46% (*v*/*v*)] to modulate ScENT1′s substrate transport profile is expected to be biologically relevant by increasing cytosolic nucleoside/nucleobase concentrations during elevated metabolic stress (e.g., early stationary phase of fermentation). Laboratory strains of *S. cerevisiae* have low to moderate levels of ethanol tolerance (e.g., 6–12% (*v*/*v*) ethanol) while natural or industrial strains can accommodate ethanol concentrations ranging from 16% to 20% (*v*/*v*) [38]. Under fermentation conditions, *S. cerevisiae* cells undergo distinctive metabolic stages during cell growth (latent, exponential growth, early-stationary, and stationary) [39,40]. The early-stationary phase is of particular note since this is when cells reach their highest density until available nitrogen sources are depleted. As fermentation slows, sugar is converted into ethanol which, ultimately, leads to autophagy at elevated ethanol concentrations and depleted metabolic precursors (e.g., nucleosides/nucleobases) [41]. A key component to autophagy is activation of ribosome degradation with targeting of ribosomal RNAs to the vacuole where they are catabolized into individual nucleosides and nucleobases [42]. We hypothesize that ScENT1 mediates flux of these nucleosides/nucleobases back to the cytoplasmic pool to aid in reestablishing metabolic balance. Our results support this hypothesis in that ScENT1 substrate transport profile shifts in the presence of alcohols (Figure S5a). In the absence of alcohols (–OH) ScENT1 is capable of low level UTP transport (Figure 6). One possible explanation for the phenomenon is that, during the exponential growth phase, ScENT1 may export UTP from the vacuole to be utilized by CTP synthetase. CTP synthetase is a cytosolic associated amidotransferase that catalyzes transfer of an amide nitrogen from glutamine to UTP in order to form CTP [43]. CTP synthetase is essential in the synthesis of all membrane phospholipids for eukaryotic cells [44]. Under conditions of ethanol stress and nitrogen starvation, phospholipid synthesis is presumably altered since *S. cerevisiae* is known to have an altered membrane composition to promote ethanol tolerance [41].

The mean substrate transport of Native-ScENT1, and F249I mutants, were reduced when compared to assays prepared using our previously reported conditions [12] (Figure S5a,b). Interestingly, the L390A mutant was less affected by alcohol relative to the F249I mutant or wildtype constructs (Figure S5c). These data may suggest, when combined with structural implications of L390, that ethanol interacts with the translocation pore in a conformation dependent manner. The odorant binding protein LUSH from *Drosophila melanogaster* has been previously shown to bind alcohol through a set of concerted hydrogen bonding interactions [45]. Hydrogen bonding networks coupled to substrate transport have also been previously observed in numerous MFS transporters, including LacY [46]. The concentrations of 2-propanol and β-mercaptoethanol were 130 mM and 5 mM, respectfully, prior to membrane insertion and purification by SEC—therefore, the final concentrations of each is negligible. However, minimal concentrations of alcohols possessing higher lipid solubilities may still increase membrane fluidity without decreasing integrity, thus lowering activation barriers and allowing ScENT1 to have more conformational flexibility. While alcohols have been associated with promoting secondary structure [47,48,49], they more commonly act as protein denaturants. An alternative explanation for the observed alcohol effects is that alcohols induce a non-native conformation, which alters substrate specificity and transport, by reducing available hydrogen bonding interactions through dehydration of solvation shells surrounding exposed amino acids. 

One of the main hurdles that industrial fermentation using *S. cerevisiae* must overcome is ethanol induced toxicity. The mechanisms of ethanol tolerance remain poorly understood. Ethanol is known to modulate cellular transport systems [50], cell metabolism, and biosynthesis of macromolecules [51]. From a physical perspective, ethanol directly impacts membrane composition and fluidity and protein (both hydrophobic and hydrophilic) conformation and stability [52]. The observation that ethanol attenuates ScENT1-mediated uridine transport is consistent with previous observations on other yeast systems and proteins. For instance, ethanol alters yeast amino acid permease, itself an IMP, function and activity [53]. Additionally, systematic mutation sets, in S288C ScENT1-null cells, decrease resistance to ethanol induced stress [54] and decrease utilization of glutamine, a key nitrogen source in metabolically active yeast [55]. In addition to systematic mutation sets, classical genetics using S288C ScENT1-null cells demonstrate that NAD^+^ accumulation decreases [56] and *N*-ribosylnicotinamide (NmR) accumulation and excretion is increased [14]. Furthermore, ScENT1 contributes to the redox state of *S. cerevisiae* by transporting NmR [14,56], a nucleoside precursor of NAD^+^. Given that ScENT1 is suspected to be a regulator of metabolic precursors contained within the vacuole by maintaining concentrations of cytoplasmic pools, we hypothesize that ScENT1 is capable of sensing ethanol and modulating its transport capabilities in response to ethanol induced stress.

Human ENTs, specifically hENT1, have also been associated with ethanol inhibition [19,21,57], preference for the consumption of ethanol [8], and SNPs associated with alcohol withdrawal seizures [7]. The ability of hENT1 to regulate adenosine flux, has recently been shown to affect glutamate release, thus regulating glutamatergic neurotransmission [6] while hENT1 inhibitors were shown to attenuate seizure severity [6]. In light of these recent findings, hENT1 may play a role in epilepsy and seizure activity associated with alcohol withdrawal syndrome. Indeed, genetic polymorphisms of hENT1 are associated with alcoholism and an increased risk of alcohol withdrawal seizures [7]. ENT1 knockout mice have increased voluntary ethanol self-seeking behaviors associated with increased resistance to acute ethanol intoxication and reduced aversive effects of ethanol [18,58]. Mutational analysis has also identified residue I216 in hENT1 as contributing to ethanol sensitivity [59]. Ethanol was previously demonstrated to reduce gemcitabine cytotoxicity in HTB2 cells, which are derived from a human papilloma [17]. hENT1 protein expression level is strongly correlated with patient outcomes and drug sensitivity in cancer therapies that utilize nucleoside analogs such as cytarabine (e.g., leukemia or lymphoma) or gemcitabine (e.g., pancreatic or lung cancer) [4,60,61,62,63,64,65,66,67]. A definitive correlation is unknown though hENT1-dependent (e.g., inhibition of hENT1 function or modulation of hENT1 expression level) and hENT-independent (e.g., attenuated deoxycytidine kinase activity, increased MDR1 activity, RRM, etc.) mechanisms have been proposed. 

## 4. Materials and Methods

### 4.1. Molecular Cloning and Protein Expression

Native FUN26 and single point mutants (L390A and F249I) were cloned as described previously (12). Protein expression utilized W303-Δpep4 (*leu2-3*, *112trp1-1*, *can1-100*, *ura3-1*, *ade2-1*, *his3-11*, *15 Δpep4MATα*) *S. cerevisiae* cells that were transformed using sheared salmon sperm DNA*.* Yeast cells positive for the native FUN26, L390A, or F249I transformants were grown in 1× synthetic complete histidine dropout media (SC-His) containing 1× Complete Supplement Mixture minus histidine (CSM-His, Sunrise Science Products, San Diego, CA, USA, catalog No. 1006), 0.67% (*w*/*v*) yeast nitrogen base without amino acids, 1% (*w*/*v*) glucose, and 1% (*w*/*v*) raffinose. Cultures were grown in 10 L working volumes using a 12 L fermenter at 30 °C with 500 μL of Antifoam 204. Agitation was set from 200 to 350 rpm based on a dissolved oxygen range of 90% to 20% with an airflow rate of 2.5 L/min Each round of protein expression contained 7.125 L of 1× SC-His media, and 375 mL of overnight culture. Following 24 h of growth, protein expression was induced by adding 2.5 L of yeast extract-peptone-galactose containing 8% (*w*/*v*) yeast extract, 16% (*w*/*v*) peptone, and 8% (*w*/*v*) galactose. After induction, cells were grown for 16 h at 30 °C using the same dissolved oxygen based agitation scheme described above. Airflow was increased to 5.0 L/min post induction. Cells were harvested by centrifugation at 3600× *g* for 30 min at 4 °C. 

### 4.2. Membrane Preparation, Solubilization and Purification

Native ScENT1 and corresponding mutants, were purified following a previously published detailed protocol (12). Briefly, cells were disrupted at ~28,000 psi using an Avestin C-3 Emulsiflex, membranes were collected by ultra-centrifugation (101,000× *g*), and solubilized using ~9 mM *N*-dodecyl-β-d-maltoside (DDM). Proteins were initially purified using TALON cobalt resin (Pierce, catalog No. 89965) followed by size exclusion chromatography on a Superdex 200 10/300 (GE Healthcare, Little Chalfont, UK, catalog No. 17-5175-01) column. Alcohol free preparations (–OH) were obtained by the removal of DHALT and PMSF protease inhibitors, and β-mercaptoethanol at all steps from cell harvest through final protein preparations. ScENT1 preparations were validated using SDS-PAGE, western blotting using an anti-poly histidine tag (Millipore Sigma, Burlington, MA, USA, catalog No. AB3517), and sequencing of protein digests using mass spectrometry. 

Preparation of proteoliposomes (PLs) containing FUN26 and empty liposomes has been described previously [12]. Assays denoted as +OH utilized protein preparations containing residual alcohols from protease inhibitor cocktails (DHALT or PMSF) and β-mercaptoethanol while, –OH denotes their absence. Carboxyfluorescein (CF)-containing liposomes and PLs were prepared by resuspending the lipid mixture (28.25 mg *E. coli* polar lipids and 1.6 mg chicken egg l-α-phosphatidylcholine per mL of buffer) with 50 mM KH_2_PO_4_ pH 7.4 buffer containing 50 mM CF [5(6)-carboxyfluorescein] prior to extrusion. Empty CF-liposomes and CF-PLs were then prepared and purified as previously described for the non-CF containing empty liposomes and PLs [12]. Tritium based functional assays were performed using the methods and tritiated substrates described previously [12]. Briefly, PLs are incubated with a tritiated substrate for a controlled amount of time. Substrate influx is terminated using vacuum filtration. Substrate uptake is observed by the presence of radioactive signal on borosilicate filters following vacuum filtration; the filters capture the PLs or empty liposomes while allowing the excess non-luminal substrate to be washed away. Specific substrate transport in pmol/mg of ScENT1 was calculated by:

Transport (pmol/mg protein) = (CPMsample−CPMcontrol)(SR × mg protein)

where CPM_sample_ are the counts on the filter associated with the proteoliposome, CPM_control_ are the counts on the filter associated with the empty liposomes, and SR is the specific radioactivity defined as: total radioactivity (CPM)/pmol of substrate per sample, and was described previously [68]. Ethanol inhibition was determined by pre-incubating –OH PLs (prepared by reconstituting 600 μg of ScENT1 in 200 μL of liposomes) and empty liposomes with 250 mM (ethanol or methanol) for 15 min, then incubating with 100 nM [^3^H]-uridine for 3.5 h, as previously reported for assays assessing overall substrate accumulation [12], prior to filtration. 

Fluorescence based integrity assays were performed on an ISS PC1 photon counting spectrofluorometer (Champaign, IL, USA) at 20 °C using sealed quartz cuvettes. Excitation was initiated at 492 nm and emission was monitored between 500 nm and 560 nm. Equal volumes of alcohol or water were added to each cuvette containing either CF-PLs or empty CF-liposomes. The contents of each cuvette were then mixed and capped off prior to each measurement. Alcohol concentrations ranged from 0 mM to 4.074 M. 

### 4.3. Gaussian Modeling

MarvinSketch version 6.0.3 (ChemAxon, Cambridge, MA, USA) was used to generate 2D models and Cartesian coordinate files for each substrate. The coordinate files were then cleaned using GaussView 5 (Gaussian, Wallingford, CT, USA) and normalized based on the ribose ring prior to being modeled with Gaussian 09 (Gaussian, Wallingford, CT, USA). All substrates were considered neutral with a spin multiplicity of 1. Substrates modeled using Gaussian 09 underwent a tight geometric optimization at a MP2/6-31G** theory level followed by an energy optimization with a full population analysis at the same theory level using both non-solvated and fully water solvated constraints. 

### 4.4. Ab Initio Model of the ScENT1 Inward-Open Configuration

Rosetta version 3.4 (Rosetta Commons, www.rosettacommons.org) *ab initio* modeling software was used to generate an MFS-anchored comparative structural model of ScENT1 [69]. Structure fragments were generated using the SAM-T99 and PSI-PRED secondary structure prediction methods. Twenty thousand independent structures were generated and subjected to clustering analysis and binning. Centroids of the three largest clusters were selected with α-carbon backbones compared relative to the outward-open FucP transporter structure (PDB ID: 3O7P) [70]. The top three structures represented minimal relative deviation to each other and identical membrane topology as predicted using TMPred [71] and described previously for other ENTs [3]. The most representative Rosetta model was also compared to models generated using MODELLER [72] and LOMETS [73] servers which also produced inward-open models with similar topology to our Rosetta fragment-based ab initio model. 

### 4.5. Statistical Analysis

The significance of mean substrate transport (pmol substrate/mg of ScENT1) by the L390A and F249I mutants relative to native construct was determined by two-way analysis of variance and Dunnett’s multiple comparison test (Figure 1 and Figure S6) relative to [^3^H]-cytarabine uptake into Native PLs. The significance of alcohol free substrate transport (Figure 6) was determined by comparing mean substrate uptake (pmol substrate/mg of ScENT1) relative to [^3^H]-cytarabine by ScENT1 using one-way analysis of variance and Dunnett’s multiple comparison test. Significance between –OH and +OH substrate transport assays were made by comparing mean substrate uptake (pmol substrate/mg of ScENT1) of the alcohol free assays to the assays performed in their presence by using multiple unpaired *t*-test not corrected for multiple comparisons, consistent standard deviations were not assumed, and alpha was equal to 5.000% (Figure S5a–c). Kinetic studies (e.g., V_max_ and K_m_) were fitted directly using nonlinear regression for each individual experiment (Figure 2). Ethanol and methanol inhibition of ScENT1-mediated transport was determined using a one-way analysis of variance and Dunnett’s multiple comparison tests (Figure 4). All statistical analysis was conducted using version 6.03 of GraphPad PRISM 6 for Mac (GraphPad, La Jolla, CA, USA) and Microsoft Excel version 14.0.7106 (Microsoft Corporation, Redmond, WA, USA). 

## 5. Conclusions

ScENT1 (formerly known as FUN26) is the *S. cerevisiae* ortholog to hENT1-3 and mutagenesis studies have analyzed the function of residues conserved throughout various members of the ENT family. We developed an ab initio model that is consistent with prior models of protozoan ENTs [27,28], and other members of the MFS [10]. While ScENT1 has a unique transport profile relative to other ENTs, it contains conserved elements with similar ENT functions—suggesting similar structural arrangements. Additionally, we show that like hENT1, ScENT1-mediated transport is capable of being attenuated in the presence of ethanol. A clear relationship between ENT function and cancer therapy or mechanistic causes of human disease remain elusive in the absence of definitive structure-function insights [74,75,76]. The current study correlates transport activity with sidechain identity for a small subset of highly conserved amino acids within the ENT family. These data further support, using purified protein, an argument that known human SNPs (e.g., G463 or G216) abrogate ScENT1 transport activity and that presence of specific ENT mutations may be viable screens to guide treatment protocols utilizing nucleoside analog therapeutics. Furthermore, follow-on studies to determine if ScENT1 functions as an ethanol sensor to modulate metabolite transport in response to ethanol induced stress are needed.

## Supplementary Materials

The following are available online. Initial uptake rates for [^3^H]-cytidine, [^3^H]-uridine, and [^3^H]-UTP showing linearity across 60 min for wildtype (WT), F249I, and L390A ScENT1 are show in Figure S1. A multiple sequence alignment between ScENT1, human ENTs 1–3, and LdNT1.1 is shown in Figure S2. Electrostatic surface potentials of tested substrates with C(2′) modifications were calculated using Gaussian 09 and shown in Figure S3. Proteoliposome integrity was tested by adding ethanol (0–4.074 M) or equal volumes of water to CF loaded proteoliposomes (a) or empty liposomes (b) and the resultant emission spectra are shown in Figure S4. Figure S5 highlights the differences in transport profiles between alcohol and alcohol-free preparations for Native ScENT1 (a) and the F249I (b) and L390A (c) mutants. Figure S6 compares radiolabeled substrate uptake between Native ScENT1 and the L390A and F249I mutants in the absence of alcohol. Table S1 displays the calculated mulliken charges on the C(2′) carbon of tested substrates with C(2′) modifications.

## Abbreviations

ENT	Equilibrative nucleoside transporter
FUN26	Function unknown now 26
ScENT1	*Saccharomyces cerevisiae* equilibrative nucleoside transporter 1
IMP	Integral membrane protein
FDA	Food and Drug Administration
EMA	European Medicines Agency
hENT	Human equilibrative nucleoside transporter
TMD	Transmembrane domain
MFS	Major facilitator superfamily
PL	Proteoliposome
SC-His	Synthetic complete histidine dropout media
DDM	*n*-Dodecyl-β-d-maltoside
CF	5(6)-Carboxyfluorescein
LacY	Lactose permease
GlpT	Glycerol 3-phosphate transporter
PHID syndrome	Pigmentary hypertrichosis and non-autoimmune insulin-dependent diabetes mellitus
Mhp1	MAP-Homologous Protein 1
LdNT1.1	*Leishmania donovani* nucleoside transporter 1.1
LUSH	General odorant binding protein lush
SEC	Size exclusion chromatography
NmR	*N*-ribosylnicotinamide
ns	Not significant
