# Supplementary material for: Functional Characterization of the Saccharomyces cerevisiae Equilibrative Nucleoside Transporter 1 (ScENT1)
Source: Molecules. 2018 Mar 22;23(4):732. doi: 10.3390/molecules23040732 (PMC6017673; doi:10.3390/molecules23040732)
Supplement: Supplementary file 1 [file molecules-23-00732-s001.pdf]

Supporting Information (Boswell-Casteel, et al.)

Figure S1: Time-dependent uptake into Native and mutant ScENT1 proteoliposomes. Shown here are representative assays of time-dependent uptake of (a) [^{3}H]-uridine, (b) [^{3}H]-cytidine, and (c) [^{3}H]-UTP using 100 nm and 2000 nm substrate concentrations. Uptake was determined at 10, 20, 50, and 60 minutes. A linear fit to each data set was obtained using a nonlinear regression model with R^2 values listed in the figure legends (0,0 was not included in the fitting). 100 nM L390A (green), 2000 nM L390A (orange), 100 nM Native (WT, blue), 2000 nM Native (WT, magenta), 100 nM F249I (cyan), and 2000 nM F249I (red).

Figure S2: Multiple sequence alignment with ScENT1 and other ENTs. ScENT1 was aligned with other human ENTs 1-3 (hENT) and the *Leishmania donovani* (LdNT1.1) using the PROMALS3D webserver.[31, 32] Strictly conserved residues are denoted in red and bolded, similar residues as determined by PROMALS3D are depicted in blue, the location of the ScENT1 TMDs are denoted by a string of T's, and mutated residues discussed in this article are boxed in.

Figure S3: Electrostatic surface potentials of substrates relevant to C(2`) modifications. Electrostatic surface potentials were calculated using Gaussian 09 and mapped onto the noted substrates. Electrostatic surface potentials were normalized and ranged from - 0.09979 to 0.113 with lower potential values being represented by the cooler end of the spectrum (blue) and higher potential values are shown with warmer colors (red).

Figure S4: Membrane integrity is maintained over a broad range of ethanol concentrations. Membrane integrity of CF loaded PLs (a) and empty liposomes (b) were tested by adding increasing concentrations of ethanol and collecting emission spectra to check for an increase in fluorescence signal that would signify permeabilization of the artificial membrane and release of CF. Ethanol concentrations ranged from 0 mM (green trace) to 4.074 M (red trace). The 517 nm (blue line) and 525 nm (red line) wavelengths are marked for a visual reference.

Figure S5: Effects of residual alcohols on radiolabeled substrate uptake. Substrates have been classified based on containing a pyrimidine or purine nucleobase and data represents the mean substrate uptake (pmol substrate / mg of ScENT1) as described for Figures 1 and 6, each substrate was tested at 100 nM final concentration in the assay sample mixture prepared in the absence of any residual alcohols. PLs were incubated with radiolabeled substrate for 3.5 hours followed by vacuum filtration onto membranes. Error represents the S.E.M. Statistical significance was determined by comparing mean substrate uptake of the (+ OH) preps to the (- OH) preps for Native (a), F249I (b), and L390A (c) using multiple unpaired t-test not corrected for multiple comparisons, consistent standard deviation was not assumed, and alpha was equal to 5.000%. Statistically significant changes are denoted in red (decrease) or green (increase). . ns, not significant; *, $p < 0.05$; **, $p < 0.01$; ***, $p < 0.001$; ****, $p < 0.0001$.

Figure S6: Comparison of radiolabeled substrate uptake between Native ScENT1 and mutants in the absence of alcohol. Substrates have been classified based on containing a pyrimidine or purine nucleobase and data represents the mean substrate uptake (pmol substrate / mg of ScENT1) from no less than N=3 independent observations for each substrate tested at 100

nM final concentration in the assay sample mixture prepared in the absence of any residual alcohols. PLs were incubated with radiolabeled substrate for 3.5 hours followed by vacuum filtration onto membranes. Error bars represent the S.E.M. Statistical significance of the L390A and F249I PLs was determined by comparing the mean substrate uptake for each individual substrate to the mean substrate uptake of the respected Native PLs using two-way analysis of variance and Dunnett's multiple comparison test. Statistically significant changes are denoted in red (decrease) or green (increase) relative to Native ScENT1. Negative control PLs and substrate specific activity are included in the pmol substrate / mg ScENT1 calculation for each sample. ns, not significant; *, $p < 0.05$; **, $p < 0.01$; ***, $p < 0.001$; ****, $p < 0.0001$.

Table S1: Mulliken charges on the C(2') carbon atom of tested substrates containing C(2') modifications. Mulliken charges were calculated using Gaussian 09 at the MP2/6-31g** theory level following a tight geometric at the same level of theory. Charges were calculated *in vacuo* and fully solvated with water, and represent only the C(2') carbon of the ribose ring.

Figure S1 (Boswell-Casteel, et al.)

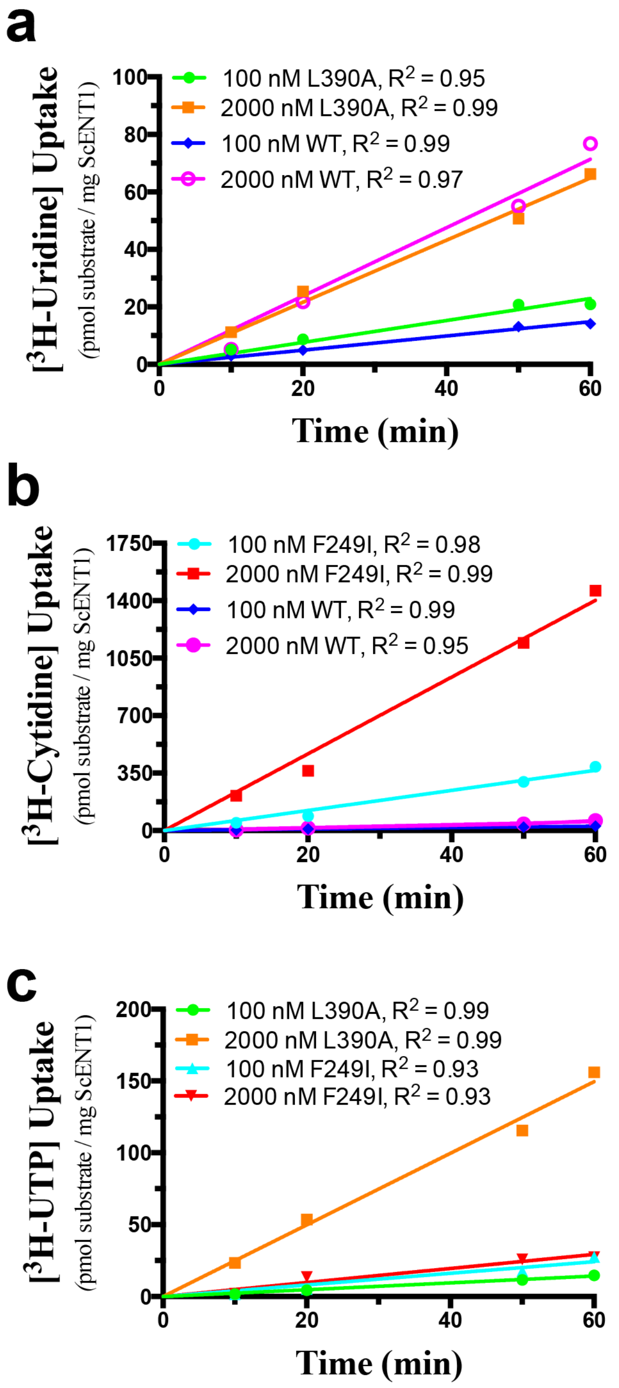

Figure S2 (Boswell-Casteel, et al.)

```
FUN26 TMDs:
FUN26          1  M---------------------------------------STSADTDTIKKPILAVPEPALADTHSEEISRSGEEHESENNEHSDEEGDNYSERE   56
hENT1          1  M---------------------------------------TTS-------------HQPQ---------------------------DRY----   11
hENT2          1  M---------------------------------------ARG-------------DAPR---------------------------DSY----   11
hENT3          1  MAVVSEDDFQHSSNSTYRTTSSSLRADQEALLEKLLDRPPPGL-------------QRPE---------------------------DRF----   50
LdENT1.1       1  M---------------------------------------DTAPDH----------REPQ----------------------------EQGESRK   18

FUN26 TMDs:                                  TTTTTTTTTTTTTTTTTTTTTTT                                        TTTTTTT
FUN26         57  QSVSTEPLDTLPLRKKL---KNLSYITFFAIGIGLLWPWNCILSASQYFKHDIFKDT--------------------------SIWAKIFTSSMM  122
hENT1         12  ---------------------KAVWLIFFMLGLGTLLPWNFFMTATQYFTNRLDMSQNVSLVTAELSKDAQASAAPAAPLPERNSLSAIFNNVMT   85
hENT2         12  ---------------------HLVGISFFILGLGTLLPWNFFITAIPYFQARLAGAGNSTARILSTN-------------HTGPEDAFNFNNWVT   72
hENT3         51  ---------------------CGTYIIFFSLGIGSLLPWNFFITAKEYWMFKLRNSSSPATGED----------------PEGSDILNYFESYLA  108
LdENT1.1      19  W-------------YEMTASEFYVYVVAFMCGVSMMMPVNAVFSAPAYIMTYYRYAMQDPEAVPLY--------------TNFWNNVMTYYNLIG   86

FUN26 TMDs:       TTTTTTTTTTTTTTT              TTTTTTTTTTTTTTTTTTTTTTT      TTTTTTTTTTT TTTTTTTTTTTT            T
FUN26        123  SFSTISSMLFNIYLAKRQYKYS--RRVINGLVWEIIVFTVMCFFTILHFLLPK--WFNFMFIMMLVVIS-SMGTAMTQNGIMAIANVFGSEYSQG  212
hENT1         86  LCAMLPLLLFTYLNSFLHQRIPQSVRILGSLVAILLVFLITAILVKVQ--LDA--LPFFVITMIKIVLI-NSFGAILQGSLFGLAGLLPASYTAP  175
hENT2         73  LLSQLPLLLFTLLNSFLYQCVPETVRILGSLLAILLLFALTAALVKVD--MSP--GPFFSITMASVCFI-NSFSAVLQGSLFGQLGTMPSTYSTL  162
hENT3        109  VASTVPSMLCLVANFLLVNRVAVHIRVLASLTVILAIFMVITALVKVD--TSSWTRGFFAVTIVCMVIL-SGASTVFSSSIYGMTGSFPMRNSQA  200
LdENT1.1      87  IVTSLIMEPLTLLS--WFRRIPMKVRLLGGLVILIVEIIVLMVVPARG--TSE---AGAVATICCTGFIGGFGKSIFESTTYGMFGAFPSSFTST  174

FUN26 TMDs:       TTTTTTTTTTTTTTTTTTTTTT          TTTTTTTTTTTTTTTTTTTTTTT
FUN26        213  VMVGQAVAGVLPSLVLFALAFIENSS-VSTTGGILLYFFTTTLVVTICVVMFSVSKISRKVNENWNVEDGHITDVLLGSLRSNEEEIR--IVGRI  304
hENT1        176  IMSGQGLAGFFASVAMICAIASGSE----LSESAFGYFITACAVIILTIICYLGLPRL---------E--FYRYYQQLKLEGPGEQET--KLDLI  253
hENT2        163  FLSGQGLAGIFAALAMLLSMASGVD----AETSALGYFITPCVGILMSIVCYLSLPHL---------K--FARYYLANKSSQAQAQELETKAELL  242
hENT3        201  LISGGAMGGTVSAVASLVDLAASSD----VRNSALAFFLTATVFLVLCMGLYLLLSRL---------E--YARYYMRPVLAAHVFSGE-------  273
LdENT1.1     175  MMGGVGMSGVLTSLLQIIVKAALPDSYEGVKKQSKIYYGLDVGIQGMTFVALILLRFN---------S--FAQNYFGDLGAVKSKVDAGKLSAEA  258

FUN26 TMDs:                                                                                  TTTTTTTTTTTTTT TTTTTTT
FUN26        305  DQMEDEDHRRT-----------------------------------NGTRDDNDEGEELQLKVPFEVLFAKLKYLVLSIFTTFVVTL-VFPVFAS  363
hENT1        254  SKGEEPRAGK------------------------------------EESGVSVSNSQPTNESHSIKAILKNISVLAFSVCFIFTITIGMFPAVTV  312
hENT2        243  QSDENGIPSSPQKVALT------------------------LDLDLEKEPESEPDEPQKPGKPSVFTVFQKIWLTALCLVLVFTVTLSVFPAITA  313
hENT3        274  ----EELPQD------------------------------------SLSAPSVASRFIDSHTPPLRPILKKTASLGFCVTYVFFITSLIYPAICT  328
LdENT1.1     259  LCHTDEHPTHDKEGRNSSSGKEVPALGEVQTAAAKSEGPDAVEESSWPHEVEGPTSNEILVATAIFSTLRRVKWMFVACAFNFLITLFLFPGIAV  353

FUN26 TMDs:       TT             TTTTTT TTTTTTTTTTTT                      TTTTTTTTTTTTTTTTTTTTTTT         TTTTTTT
FUN26        364  ATYVTGL----PLSNAQYIPL-IFTLWNLGDIYGRVIADWPM---FRDQKFTPRKTFIYSLLRVAAIPLFLMFTAITSSSSGDEEHNGSVIVDLC  450
hENT1        313  EVKSSIAG--SSTWERYFIPVSCFLTFNIFDWLGRSLTAVFMW-----PGKDSRWLPSLVLARLVFVPLLLLCNIKPRRY-----LTVVFEHDAW  395
hENT2        314  MVTSSTSP---GKWSQFFNPICCFLLFNIMDWLGRSLTSYFLW-----PDEDSRLLPLLVCLRFLFVPLFMLCHVPQRSR-----LPILFPQDAY  395
hENT3        329  NIESLNKGSGSLWTTKFFIPLTTFLLYNFADLCGRQLTAWIQV-----PGPNSKALPGFVLLRTCLIPLFVLCNYQPRVHL----KTVVFQSDVY  414
LdENT1.1     354  GMFPDS----------KWFSTIAVFIFNVFDVLGRFSPSLKLMWPR--SYKQRWIIVAASFARVIFVPLLLLHSYH------------YIPGEAY  424

FUN26 TMDs:       TTTTTTTTTTTTTTTT                            TTTTTTTTTTTTTTTTTTTTTTT
FUN26        451  YMLLQFLFGVTNGHVISMSFMKVPEQ---LDNDDEKEAAGGFTNIFVSTGLALGSIISYVFVFIIDFIIR  517
hENT1        396  FIFFMAAFAFSNGYLASLCMCFGPKK---V-KPAEAETAGAIMAFFLCLGLALGAVFSFLFRAIV-----  456
hENT2        396  FITFMLLFAVSNGYLVSLTMCLAPRQ---V-LPHEREVAGALMTFFLALGLSCGASLSFLFKALL-----  456
hENT3        415  PALLSSLLGLSNGYLSTLALLYGPKI---V-PRELAEATGVVMSFYVCLGLTLGSACSTLLVHLI-----  475
LdENT1.1     425  GYVMEVIFGFSNGYVGSMALVLGPQSKGID-NDGKRFVAGTLMGISILVGGTIGTVLSIMTQTIRATY--  491
```

Strictly Conserved (red)
Similar Residues (blue)
TTT TMDs Loacated in FUN26
Mutated Residues (boxed)

Figure S3 (Boswell-Casteel, et al.)

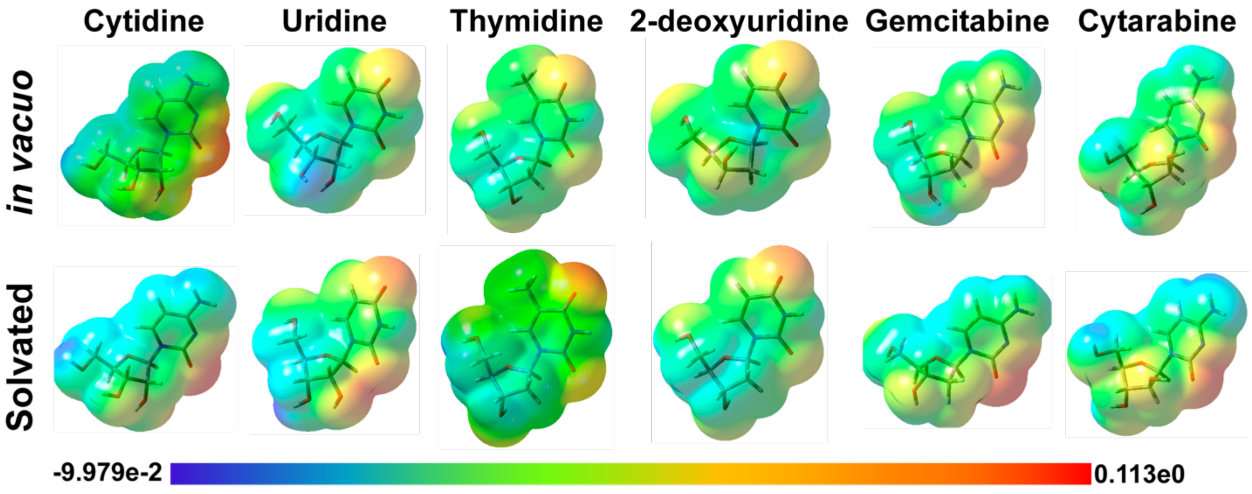

Figure S4 (Boswell-Casteel, et al.)

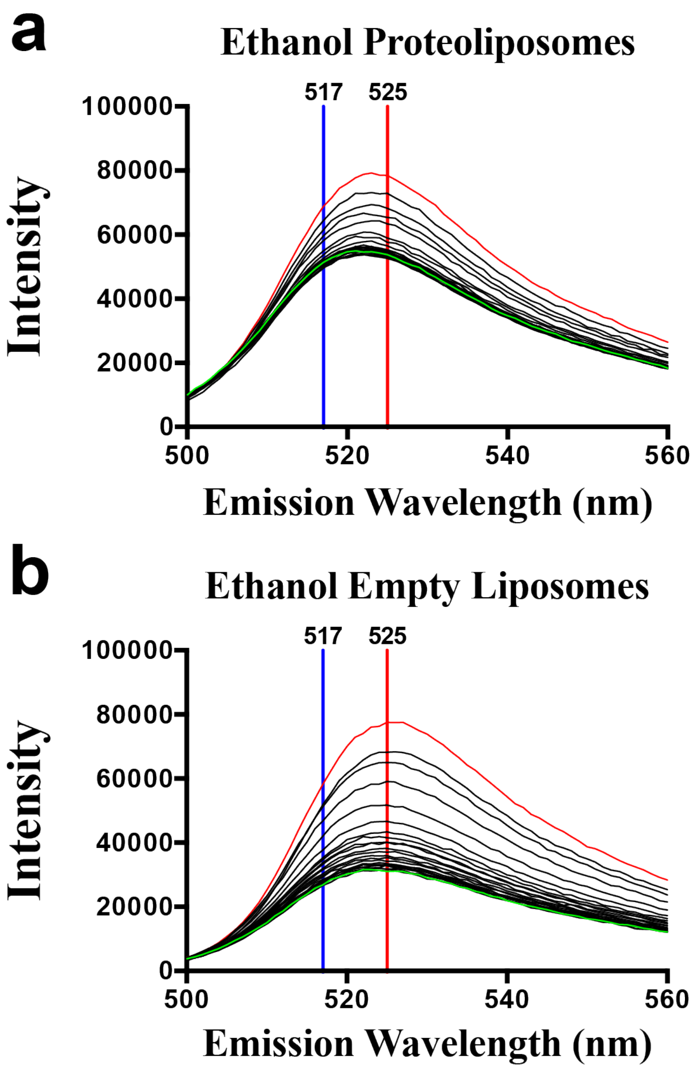

Figure S5 (Boswell-Casteel, et al.)

a Native

	Substrate	Native (+OH)	Native (-OH)	Significance
Pyrimidine	[3H]-UTP	0.354 ± 0.28	24.6 ± 3.9	(**)
	[3H]-Uridine	88.7 ± 4.7	46.9 ± 5.4	(***)
	[3H]-Cytidine	148 ± 18	66.1 ± 6.4	(***)
	[3H]-Thymidine	34.4 ± 5.0	21.6 ± 2.5	(*)
	[3H]-2-deoxyuridine	10.5 ± 3.7	46.2 ± 6.0	(***)
	[3H]-Gemcitabine	0.426 ± 0.16	0.036 ± 0.044	(*)
	[3H]-Cytarabine	0.102 ± 0.041	0.505 ± 0.22	(ns)
	[3H]-Uracil	90.7 ± 15	32.1 ± 7.6	(**)
	[3H]-Cytosine	93.2 ± 13	23.0 ± 8.8	(***)
Purine	[3H]-Adenosine	6.69 ± 1.7	39.9 ± 5.9	(**)
	[3H]-Guanosine	70.6 ± 3.6	-0.037 ± 0.14	(****)
	[3H]-Inosine	71.6 ± 8.1	21.4 ± 4.4	(****)
	[3H]-Adenine	133 ± 16	69.4 ± 9.8	(**)
	[3H]-Hypoxanthine	50.2 ± 2.6	67.5 ± 6.5	(ns)

b F249I

	Substrate	F249I (+OH)	F249I (-OH)	Significance
Pyrimidine	[3H]-UTP	70.3 ± 2.2	47.4 ± 5.9	(*)
	[3H]-Uridine	172 ± 7.9	67.4 ± 4.1	(***)
	[3H]-Cytidine	192 ± 29	72.3 ± 2.0	(*)
	[3H]-Thymidine	70.5 ± 4.5	18.5 ± 1.1	(***)
	[3H]-2-deoxyuridine	154 ± 25	38.0 ± 2.2	(**)
	[3H]-Gemcitabine	0.197 ± 0.12	1.24 ± 0.22	(**)
	[3H]-Cytarabine	-0.0460 ± 0.027	0.806 ± 0.10	(***)
	[3H]-Uracil	73.7 ± 7.9	30.4 ± 1.1	(**)
	[3H]-Cytosine	152 ± 19	72.4 ± 6.9	(*)
Purine	[3H]-Adenosine	84.0 ± 10	92.6 ± 2.8	(ns)
	[3H]-Guanosine	26.8 ± 1.8	25.2 ± 0.74	(ns)
	[3H]-Inosine	70.9 ± 1.5	58.3 ± 1.1	(**)
	[3H]-Adenine	157 ± 7.2	181 ± 4.3	(*)
	[3H]-Hypoxanthine	179 ±4.4	164 ± 6.9	(ns)

c L390A

	Substrate	L390A (+OH)	L390A (-OH)	Significance
Pyrimidine	[3H]-UTP	56.3 ± 3.6	90.6 ± 1.6	(***)
	[3H]-Uridine	62.4 ± 4.9	78.3 ± 4.2	(ns)
	[3H]-Cytidine	89.0 ± 12	92.1 ± 8.1	(ns)
	[3H]-Thymidine	58.9 ± 2.0	43.4 ±1.5	(**)
	[3H]-2-deoxyuridine	92.8 ± 15	46.2 ± 1.9	(ns)
	[3H]-Gemcitabine	0.0203 ± 0.049	0.625 ± 0.25	(*)
	[3H]-Cytarabine	0.140 ± 0.036	0.168 ± 0.087	(ns)
	[3H]-Uracil	115 ± 18	4.89 ± 0.312	(**)
	[3H]-Cytosine	33.3 ± 4.4	38.6 ± 0.44	(ns)
Purine	[3H]-Adenosine	29.4 ± 3.2	44.7 ± 2.3	(*)
	[3H]-Guanosine	16.3 ± 3.0	0.605 ± 0.080	(**)
	[3H]-Inosine	55.4 ± 6.0	25.2 ± 3.4	(**)
	[3H]-Adenine	73.1 ± 15	72.7 ± 7.9	(ns)
	[3H]-Hypoxanthine	45.0 ± 14	11.9 ± 9.8	(ns)

Figure S6 (Boswell-Casteel, et al.)

	Substrate	Native (-OH, N = 9)	L390A (-OH, N = 3)	F249I (-OH, N = 3)
Pyrimidine	[3H]-UTP	24.6 ± 3.9	90.6 ± 1.6 (****)	47.4 ± 5.9 (*)
	[3H]-Uridine	46.9 ± 5.4	78.3 ± 4.2 (**)	67.4 ± 4.1 (ns)
	[3H]-Cytidine	66.1 ± 6.4	92.1 ± 8.1 (*)	72.3 ± 2.0 (ns)
	[3H]-Thymidine	21.6 ± 2.5	43.4 ±1.5 (*)	18.5 ± 1.1 (ns)
	[3H]-2-deoxyuridine	46.2 ± 6.0	46.2 ± 1.9 (ns)	38.0 ± 2.2 (ns)
	[3H]-Gemcitabine	0.036 ± 0.044	0.625 ± 0.25 (ns)	1.24 ± 0.22 (ns)
	[3H]-Cytarabine	0.505 ± 0.22	0.168 ± 0.087 (ns)	0.806 ± 0.10 (ns)
	[3H]-Uracil	32.1 ± 7.6	4.89 ± 0.312 (*)	30.4 ± 1.1 (ns)
	[3H]-Cytosine	23.0 ± 8.8	38.6 ± 0.44 (ns)	72.4 ± 6.9 (****)
Purine	[3H]-Adenosine	39.9 ± 5.9	44.7 ± 2.3 (ns)	92.6 ± 2.8 (****)
	[3H]-Guanosine	-0.037 ± 0.14	0.605 ± 0.080 (ns)	25.2 ± 0.74 (*)
	[3H]-Inosine	21.4 ± 4.4	25.2 ± 3.4 (ns)	58.3 ± 1.1 (***)
	[3H]-Adenine	69.4 ± 9.8	72.7 ± 7.9 (ns)	181 ± 4.3 (****)
	[3H]-Hypoxanthine	67.5 ± 6.5	11.9 ± 9.8 (****)	164 ± 6.9 (****)

Table S1 (Boswell-Casteel, et al.)

**Mulliken Charges on the C2' Carbon**		
**Substrate**	***in vacuo***	**Solvated with H_2O**
Cytidine	0.154	0.153
Uridine	0.157	0.18
Gemcitabine	0.802	0.778
Thymidine	-0.287	-0.294
2'-deoxyuridine	-0.287	-0.295
Cytarabine	0.167	0.171
